# Supplementary material for: The Satellite DNA Catalogues of Two Serrasalmidae (Teleostei, Characiformes): Conservation of General satDNA Features over 30 Million Years
Source: Genes (Basel). 2022 Dec 28;14(1):91. doi: 10.3390/genes14010091 (PMC9859320; doi:10.3390/genes14010091)
Supplement: Supplementary file 1 [file genes-14-00091-s001.zip › Figure S1.pdf]

CmaSat03-177, PmeSat08-177

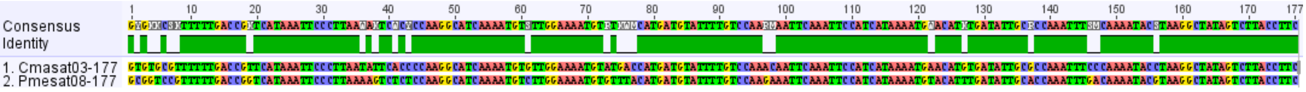

CmaSat01-144, CmaSat04-141, and PmeSat02-143

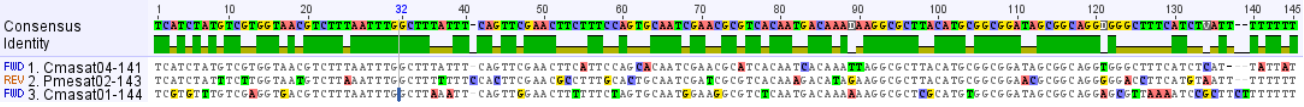

CmaSat05-247, CmaSat32-237, and PmeSat05-247

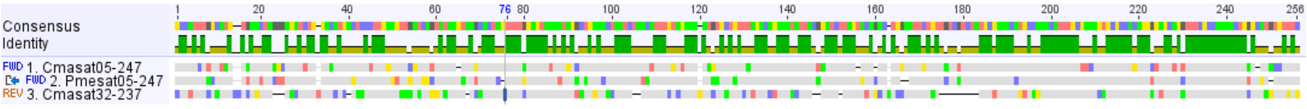

CmaSa09-42, PmeSat07-42

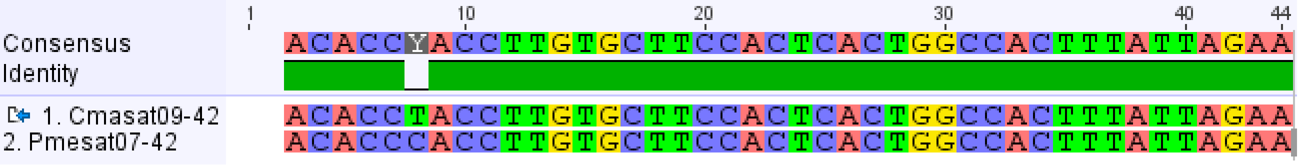

CmaSat14-170, PmeSat15-157

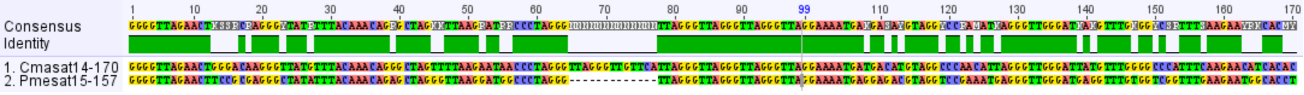

CmaSat15-21, PmeSat10-21

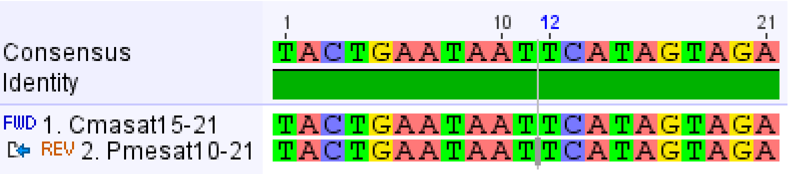

CmaSat16-955, PmeSat14-956

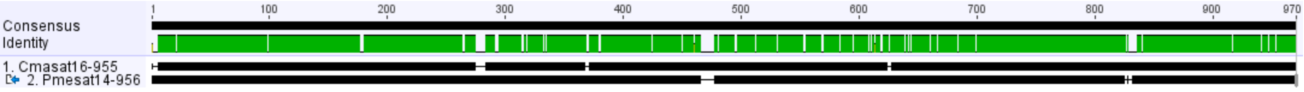

CmaSat18-30, PmeSat19-30

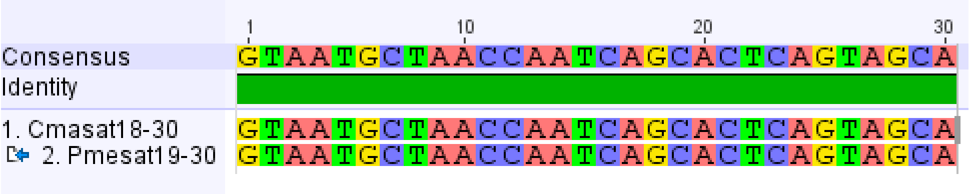

CmaSat19-38, PmeSat23-38

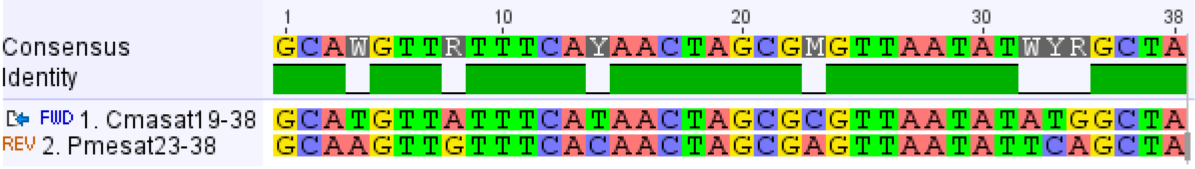

CmaSat20-72, PmeSat12-72

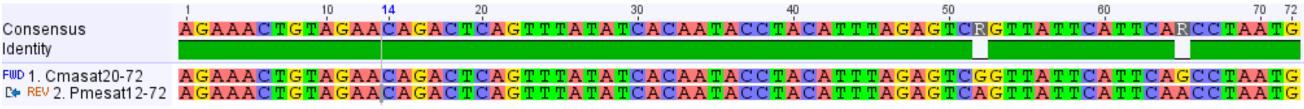

CmaSat21-28, PmeSat22-28

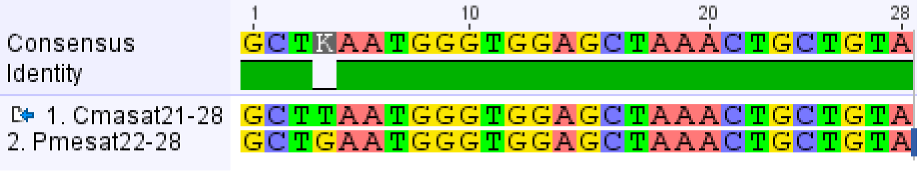

CmaSat22-68, PmeSat17-65

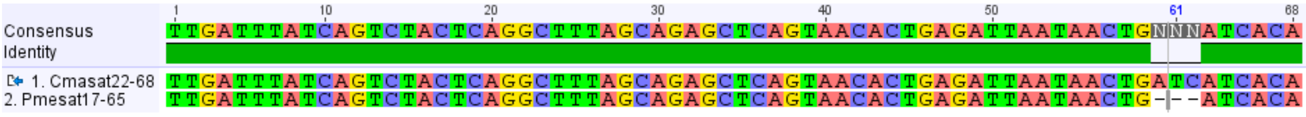

CmaSat27-394, PmeSat28-398

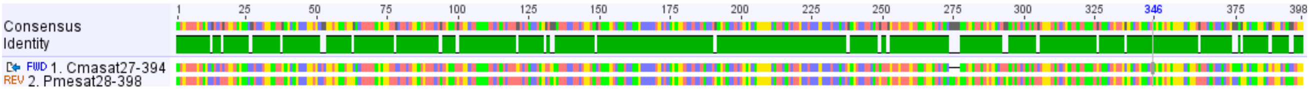

CmaSat31-54, PmeSat21-54

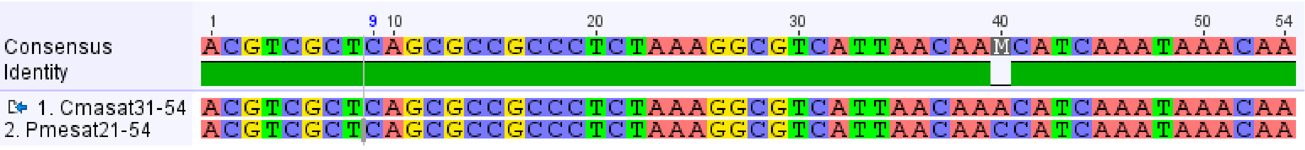

CmaSat33-66, PmeSat18-67

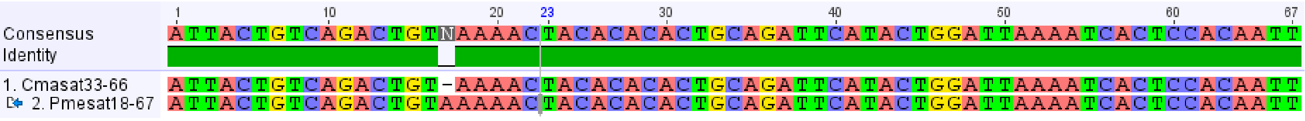

CmaSat34-101, PmeSat27-102

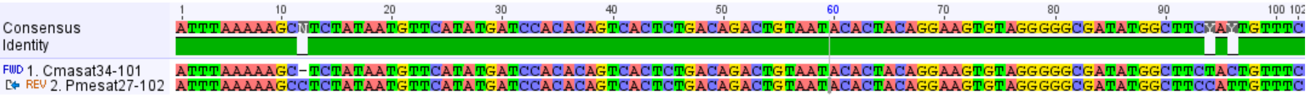

CmaSat36-1250, PmeSat11-1242

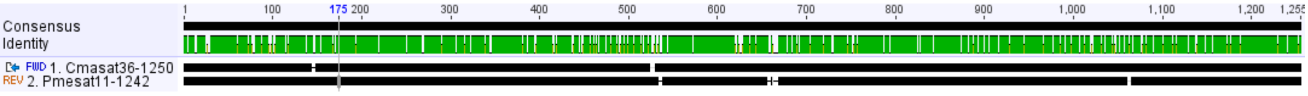

CmaSat38-30, PmeSat29-30

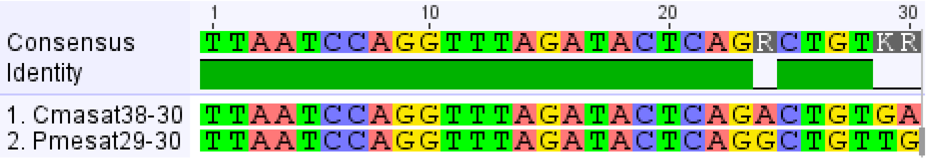

Figure S1: Alignments of shared satDNAs between *C. macropomum* and *P. mesopotamicus*.
