# Supplementary material for: The Satellite DNA Catalogues of Two Serrasalmidae (Teleostei, Characiformes): Conservation of General satDNA Features over 30 Million Years
Source: Genes (Basel). 2022 Dec 28;14(1):91. doi: 10.3390/genes14010091 (PMC9859320; doi:10.3390/genes14010091)
Supplement: Supplementary file 1 [file genes-14-00091-s001.zip › Figure S2.pdf]

CmaSat09-42, PmeSat07-42, CgoSat10-42, MmaSat07-42, PliSat12-42, TauSat06-42, MelSat08-42

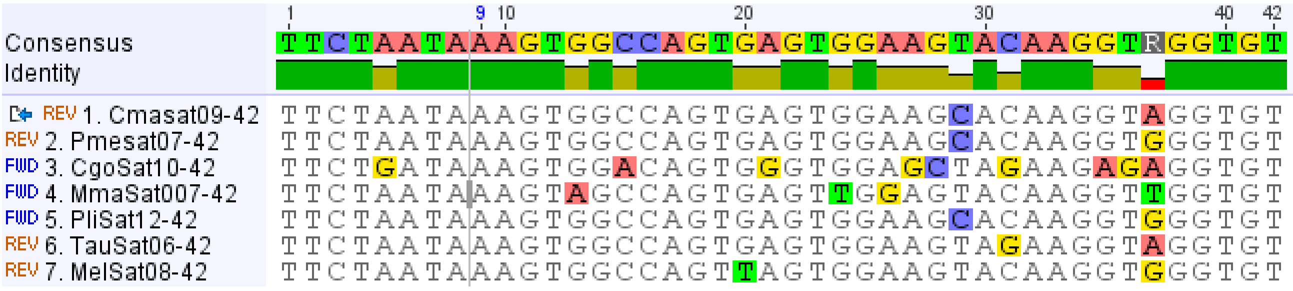

CmaSat15-21, PmeSat10-21, PliSat17-21

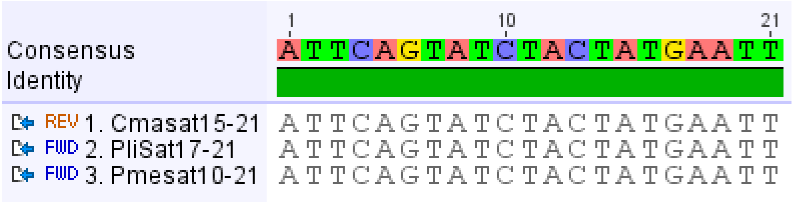

CmaSat18-30, PmeSat19-30, PliSat19-30, Mel25-30

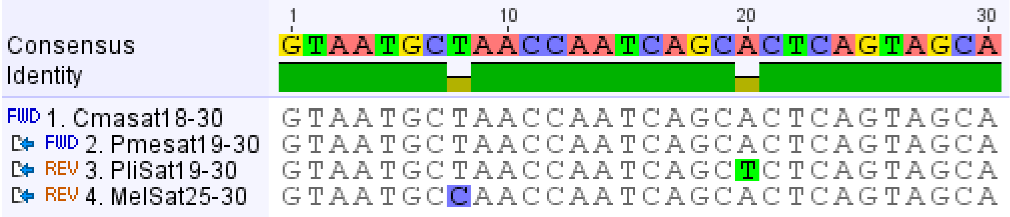

CmaSat20-72, PmeSat12-72, PliSat15-75, TauSat19-76

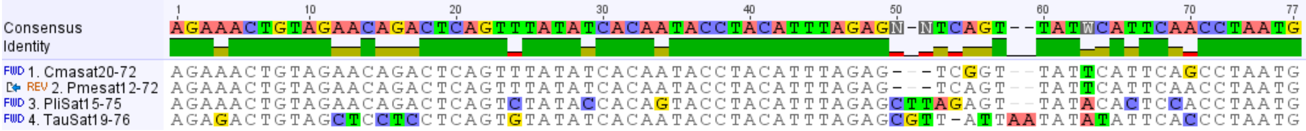

CmaSat21-28, PmeSat22-28, CgoSat26-29, MmaSat41-29, TauSat16-29, MelSat48-29

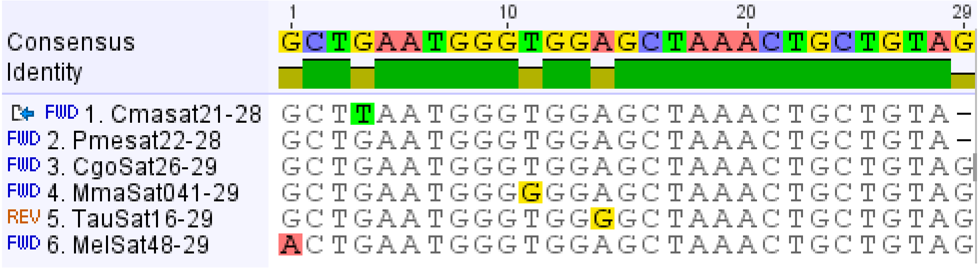

CmaSat22-68, PmeSat17-65, MmaSat84-65, PliSat36-68, TauSat12-66, MelSat39-65

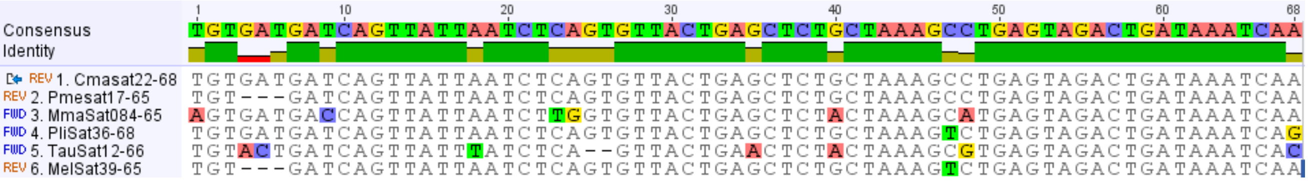

CmaSat31-54, PmeSat21-54, MmaSat38-54

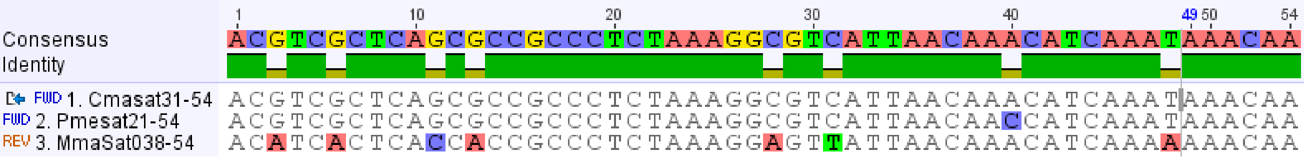

CmaSat33-66, PmeSat18-67, MmaSat27-67, PliSat16-67, MelSat12-67

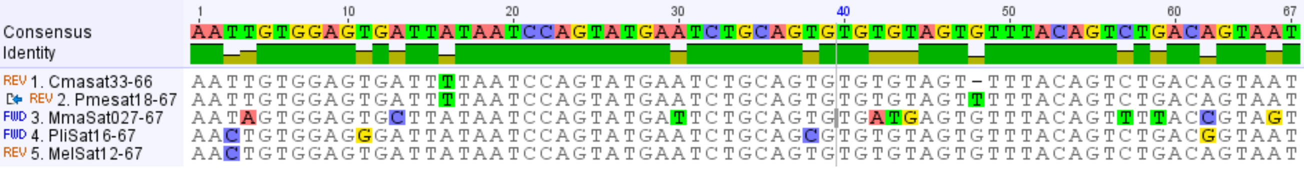

CmaSat35-30, MmaSat57-30, PliSat30-31, MelSat58-31

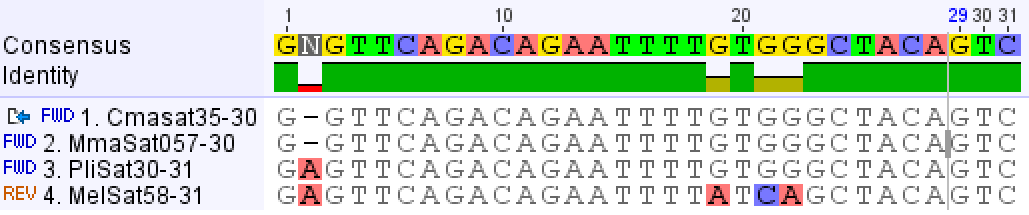

CmaSat38-30, PmeSat29-30, PliSat32-30, MelSat54-30

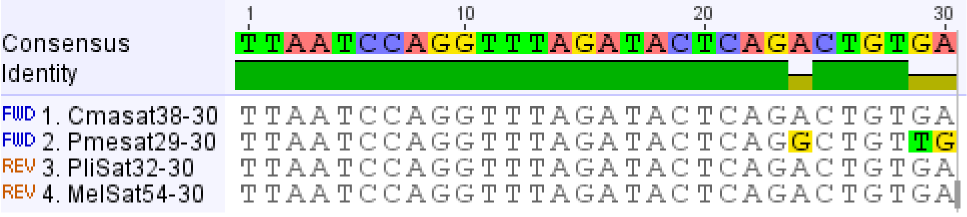

Figure S2: Alignment of SatDNA conserved between *P. mesopotamicus* or *C. macropomum* and another Characiform.
