# Supplementary material for: The Satellite DNA Catalogues of Two Serrasalmidae (Teleostei, Characiformes): Conservation of General satDNA Features over 30 Million Years
Source: Genes (Basel). 2022 Dec 28;14(1):91. doi: 10.3390/genes14010091 (PMC9859320; doi:10.3390/genes14010091)
Supplement: Supplementary file 1 [file genes-14-00091-s001.zip › Table S1.pdf]

Table S1: Designed primers in the present study.

Five most abundant satDNA in each species		
**satDNA family**	**Primer F**	**Primer R**
PmeSat01	GGGAGCGTCTTTTTGGTCAT	AGGAGCATTGGAATCCGCTT
PmeSat02	GCAAAGGCGTTCGAAGTGAA	AGGTGGACCTTCATGTAATTATTT
PmeSat03	TTTTAGCGAGAGGACACTGG	CCAGGTTCTTGTTCTCAAGC
PmeSat04	GCACTACCTCGCCTAAATCA	CCCTCTATTATCCACAGAGG
PmeSat05	GTACAATGAGCTCTCAGTAACA	CACTTGGGTGACTAGACCTG
CmaSat01	CGGCAGGAGCGTTAAAATCCG	CTATCCGCCACATGCGAGCG
CmaSat02	CTCTTAAGCCCTCTGCTTAG	CCTGAACTACTTTAGAAGGCC
CmaSat03	CCCCAAGGCATCAAAATGTG	GAACGGTCAAAAAACGCACAC
CmaSat04	CAAATTAGGCGCTTACATGCG	GTGATGCGTTCGATTGTGCTGG
CmaSat05	CAACTGAGCGCCTGTGGCA	CGCTATGTTGTATTCTCTTAACC
**Shared satDNAs**		
PmeSat08	TGTCCAAGAAATTCAAATTCCATCA	GACATTTTGATGCCTTGGAGAG
PmeSat11	GGCCTGTTTTCTTGCTAATCC	CGTTTGGCAGAGTAGATATTCT
PmeSat12	CCTAATGAGAAACTGTAGAACAG	GACTCTAAATGTAGGTATTGTGA
PmeSat14	GGATGCCCATGAAACTACCC	CAGAGTGATCATCTCTGGCAG
PmeSat15	CTTCCGCGAGGGCTATATTTA	CGACCACAAACCTCATCCCA
PmeSat17	CACTGAGATTAATAACTGATCATC	GCTAAAGCCTGAGTAGACTG
PmeSat18	ATACTGGATTAAAATCACTCCACAA	GTGTGTGTAGTTTTTACAGTCTGA
PmeSat21	CGCCCTCTAAAGGCGTCATT	GAGCGACGTTTGTTTATTTGATG
PmeSat27	GGCGATATGGCTTCTACTGT	CCTACGCTTCCTGTAGTGTA
PmeSat28	GACCACCATCCAGTGATCCA	GGCAGCATTTAACCTGGTAG
