# Supplementary figures and images for: The Satellite DNA Catalogues of Two Serrasalmidae (Teleostei, Characiformes): Conservation of General satDNA Features over 30 Million Years
Source: Genes (Basel). 2022 Dec 28;14(1):91. doi: 10.3390/genes14010091 (PMC9859320; doi:10.3390/genes14010091)

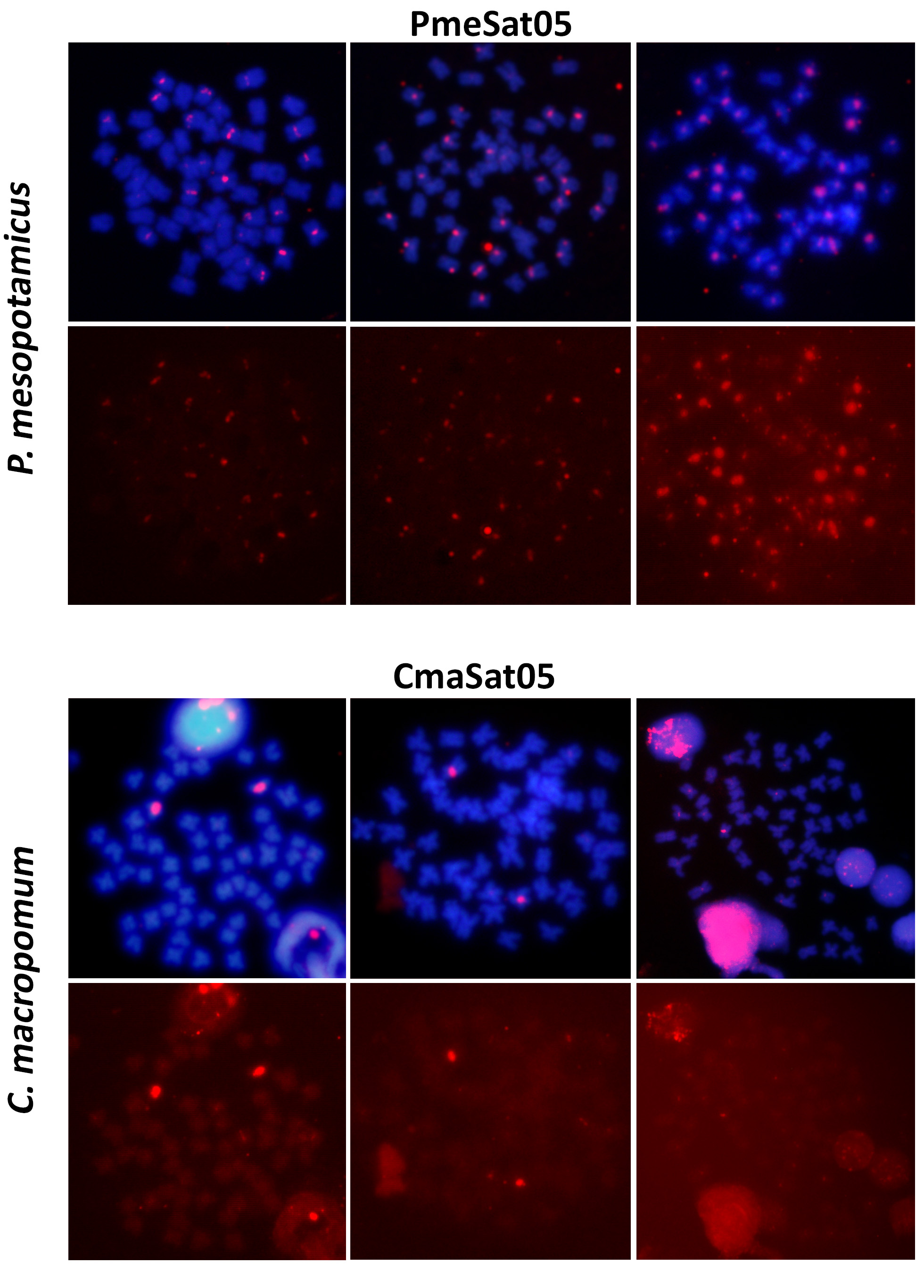

Supplement: Supplementary file 1 [file genes-14-00091-s001.zip › Figure S3.jpg]
